# Awareness of head and neck cancer among patients attended at a regional referral hospital in Tanzania

**DOI:** 10.1186/s12889-023-16333-z

**Published:** 2023-08-14

**Authors:** Zephania Saitabau Abraham, Kisula Mchele, Aveline Aloyce Kahinga

**Affiliations:** 1 Department of Surgery, School of Medicine and Dentistry, University of Dodoma, Dodoma, Tanzania; 2 Department of Otorhinolaryngology, Muhimbili University of Health and Allied Sciences, Dar Es Salaam, Tanzania

**Keywords:** Awareness, Head and neck, Cancer, Tanzania

## Abstract

**Background:**

Head and neck cancers (HNCs) are considered to be lethal and about 50% of the cases are diagnosed at advanced stages and are associated with poor prognosis. Despite the high disease burden globally, there are scarce studies on awareness of HNCs and this is the first study to explore such awareness in Tanzania. The study aimed at determining awareness of head and neck cancer among patients attended at a regional referral hospital in Tanzania.

**Methods:**

A hospital based cross sectional study was conducted at Geita Regional Referral Hospital from April to May 2022 where 315 respondents were recruited. Simple random sampling technique was utilized and data was collected using structured questionnaires and analyzed using Statistical Package for Social Sciences (SPSS) version 23. Chi-square test was performed to establish the relationship between the selected independent and dependent variables. *P*-value < 0.05 was considered to be statistically significant.

**Results:**

More than half (54.9%) of the respondents considered themselves to be somewhat knowledgeable on head neck cancer. In the same study, 56.2% of the respondents didn’t know anatomical sites of head and neck cancer and over half (65.9%) of the respondents didn't know signs and symptoms of head and neck cancer. Cigarette smoking (73.7%) and alcohol consumption (65.1%) were the most correctly identified risk factors for HNCs. Regarding treatment options and preventive measures, 75.2% of the respondents knew cessation of cigarette smoking as a preventive measure for HNCs and surgery (91.7%) was the most known treatment option for head and neck. Similarly, a significant association was found between awareness on HNCs and some of the socio-demographic characteristics of the respondents.

**Conclusions:**

Although majority of respondents considered themselves to be somewhat knowledgeable on HNCs, awareness by patients on anatomical sites, clinical features, risk factors, preventive measures and curability of head and neck cancer at the Regional Referral Hospital was minimal.

## Introduction

Head and neck cancers (HNCs) are malignant tumors occurring in various anatomical sites of the head and neck region such as nasal cavities, paranasal sinuses, nasopharynx, oral cavity, oropharynx, hypopharynx, larynx, ear, scalp, orofacial bones, thyroid and salivary glands [1, 2].

They rank the sixth among the most common cancer globally and it is estimated that 6,400,000 new cases of HNCs and 350,000 deaths occur each year worldwide [3, 4]. HNCs are associated with high morbidity and mortality due to interference with vital functions of life such as phonation, deglutition, respiration, hearing, taste and olfaction [5, 6].

Being primarily a disease of adults and aging population, head and neck cancer is four times more common in males and frequently involve African Americans [7–10]. Despite HNCs being largely preventable, the survival rate of patients with head and neck cancer has been challenging owing the anatomical location of the neoplasms [11, 12]. Since HNCs are largely preventable, public awareness remains to be of paramount importance if efforts directed at primary prevention of such cancers is to be spearheaded.

There are very limited published studies on head and neck cancer in Tanzania despite the increasing trend towards more prevalent HNCs. Available studies from Tanzania have shown a variable relative proportion of head and neck cancer where a study that was conducted at a tertiary hospital in Dar es Salaam found the relative proportion of head and neck cancer to be 3% with males (66.3%) being more affected than females (33.7%) [4]. On the other hand a study that was conducted in Mwanza at a zonal hospital found the relative proportion of head and neck cancer to be 9.5% and similarly males (67.6%)were more affected than females (32.4%) [5].

Regarding awareness on head and neck cancer, studies from different parts of the world have found most people to have inadequate awareness on HNCs despite such cancers being lethal. Studies from Saudi Arabia found 49.3% of the studied population to be unaware on HNCs and most didn’t know the symptoms for head and neck cancer where 57.8% did not recognize headache as a symptom for HNCs [13]. Similarly, a study that was conducted in the United States of America found majority of the people to be not knowledgeable on HNCs where 66.0% reported that they were “not very” or “not at all” knowledgeable on HNCs [9].

A study that was conducted in the United States of America (North Karolina) found 14% of adults to have never heard of oral or mouth cancer [14]. Similarly another study from the same country found most participants to have lower awareness on oral cancer, irrespective of their smoking or drinking habits, however smokers perceived their risk of developing oral cancer to be higher than non-smokers [15].

On the other hand, a study that was conducted in Florida found less awareness on head and neck cancers where 15.5% of adults aged 40 years and older had never heard of oral cancer and another 40.3% reportedly knew little or nothing about it. About one-half of adults did not think oral white or red patches or bleeding could indicate oral cancer [16]. Dissimilar to these findings from the USA, a study that was conducted in the United Kingdom found oral cancer to be one of the least heard cancers by the public with only 56% of the participants being aware on oral cancer [17].

There is no any published study to date on awareness of the signs, symptoms and risk factors of HNCs in Tanzania and this study aimed to address such gap by assessing awareness of head and neck cancer among patients attended a regional referral hospital in the lake zone of Tanzania.

## Methods

### Study design and study duration

It was a hospital based cross-sectional study underpinned with quantitative approach to assess awareness of head and neck cancer at Geita Regional Referral Hospital in Tanzania and data was collected from April to May 2022.

### Study setting

The study was conducted at Geita Regional Referral Hospital that is located in Lake zone. The hospital provides both in-patient and outpatient medical and surgical services to patients from various nearby regions. The hospital has several out patient clinics such as cardiology, obstetrics and gynaecology, general surgery, paediatric and child health and general outpatient clinics. Some information like percentage of patients from outpatients or inpatients or specified patients from certain specialty clinics wasn’t collected. Geita region is one of the 31 Tanzania’s administrative regions and it covers an area of 20,054 km^2^. Lake Victoria, Mwanza Region and Shinyanga Region border Geita Region to the east. The region lies between latitudes 2°8' and 3°28' South of the equator and longitudes 31° 15' and 32° 48' East of Greenwich, the Geita Region is situated in Tanzania's northern west. The region is bordered by Tabora Region and Kigoma Region to the south and south-west respectively. Lastly, Geita borders Kagera Region to the west. According to national census of 2022, the region had 2,977,608 people. Geita Region is among top 5 regions with high growth rate. The chosen regional referral hospital is not equipped with radiation therapy facility.

### Sample size calculation

Sample size for this study was calculated using the following formula:

$$\mathrm N=\frac{\mathrm Z^2\mathrm P\;\left(1-\mathrm P\right)}{\mathrm E2}$$

where;

N = Sample size

Z = Standard normal deviate = 1.96 for 95% confidence level

P = Proportion of people who are knowledgeable on head and neck cancer being taken as 24.4% [9].

E = Margin of error which is 5%

N = 283 study participants

Adjusting for non-response rate and assuming the non-response rate (f %) to be 10%; then

n′ = n x Adjusted factor

Adjusted factor = (100% / 100%-f %)

n′ = n x (100%/100%-f %)

n′ = 283 x (100%/100%-10%)

n' = 314

Therefore, the minimum sample size estimated was 314 respondents though 315 respondents were recruited in this study.

### Sampling technique and study population

A simple random sampling technique was utilized to recruit three hundred and fifteen study participants from outpatient surgical and medical departments upon consenting to participate.

### Inclusion criteria

The study population included were male and female patients aged 18 years and above who attended Geita Regional Referral Hospital both in outpatient and inpatient basis during the study period.

### Exclusion criteria

All patients with mental problems incapable of comprehending and responding to the set questions and also those who were unwilling to participate in the study were excluded.

### Recruitment methods

The principal investigator was positioned at the selected regional referral hospital and inpatients as well as those attended on outpatient basis were randomly selected until the desired sample size of 315 patients was attained.

### Data collection tools

A structured questionnaire adopted from previously published studies and thereafter modified accordingly to fit the current study was used to collect the relevant data [9, 13]. The first version was prepared in English and the final draft was translated to Swahili since the study participants in the chosen study area were more conversant with Kiswahili. The questionnaire comprised four parts: (i) socio-demographic characteristics of the study participants, (ii) Personal history of exposure to risk factors such as tobacco use/cigarette smoking, chewing tobacco, orogenital sexual practices and alcohol consumption, (iii) awareness on head and neck cancer. The questionnaire comprised both open and closed ended questions. Self-administered questionnaires were employed to collect relevant data from the selected participants. The procedure included self-introduction by the principal researcher, introduction of the topic and purpose of the study. The researcher then requested their participation in the study. Questions were asked in a manner that the sensitive questions were asked later when about to accomplish data collection from the participant. Participants were assured of free participation and withdrawal from the study at any time if they wish to do so. Moreover, reviewing the literature as well as pilot testing the instrument prior to the study by involving 10% of the actual sample size from the regional hospital and who were excluded from the actual study assessed validity of the tool.

### Measurement of variables

#### Dependent variable

The dependent variable for the study was awareness of head and neck cancer where several parameters were assessed such as whether one considers himself or herself to be knowledgeable on HNCs and this was assessed objectively by asking the study participant whether he/she consider himself/herself to be knowledgeable and no scoring criteria was utilized to assess this parameter, awareness on anatomical sites of HNCs and this was assessed using several set questions and patients were asked to choose the correct response(s) out of the set questions and similarly no scoring criteria was utilized, signs and symptoms of HNCs, risk factors for HNCs, HNCs curability and vaccines availability for prevention of HNCs, vaccine types identified suitable for prevention of HNCs, treatment options and preventive measures for HNCs and all these were similarly assessed by asking the study participants to choose the response(s) from the provided choices and no scoring criteria was utilized to assess these parameters.

#### Independent variables

The independent variables for the study were socio-demographic characteristics and smoking history of the study participants.

### Data processing and analysis

The collected data were cleaned and analyzed using SPSS version 23 software package. Descriptive statistics were performed to present frequency distribution for demographic characteristics, personal history of exposure to risk factors for head and neck cancer as well as parameters to assess awareness of head and neck cancer among the study participants.

Chi-square test was performed to establish the relationship between the selected independent and dependent variables. All the independent variables with *p*-value < 0.05 was regarded to be statistically significant.

### Ethical approval and consent to participate

The study was submitted to the Directorate of Research, Publication and Consultancy of the University of Dodoma for ethical approval. The ethical committee assessed and gave the ethical approval for this study being dated 29^th^ December 2021 under the approval number MA.84/261/02/’A’/. Furthermore, permission for conducting the research was obtained from the District Medical Officer. The individual informed consent both verbal and written was obtained from the study participants after they have been fully informed about the study goals and the process involved. The participants were ensured about privacy and confidentially. Anonymity was maintained by the use of code number on the questionnaire instead of the participant’s name and the participant had an absolute freedom and right to withdraw from the study at any time.

## Results

### Socio-demographic characteristics of the respondents

In this study, a total of 315 respondents were recruited where majority were from rural area, 175(55.6%) while those from urban area were 140(44.4%) respondents. Males, 209 (66.3%) predominated in this study and females were 106 (33.7%) (M: F = 1.94:1). Majority of the respondents belonged to the age group, 18– 33 years (52.7%) and the least number were aged ≥ 50 years, 50(15.9%). Regarding marital status, majority of the respondents were married, 256(81.3%) while 6(1.9%) were widowed. In terms of level of education, most respondents had primary level education, 169(53.7%) and 14(4.4%) had college education. (Table 1).

**Table 1:** Socio-demographic characteristics of study participants (*N* = 315)

**Variable**	**Characteristic**	**Frequency, N(%)**
**Sex**	Males	209(66.3)
	Females	106(33.7)
**Age (years)**	18– 33	166(52.7)
	34– 49	99(31.4)
	50 and above	50(15.9)
**Marital status**	Single	36(11.4)
	Married	256(81.3)
	Divorced	17(5.4)
	Widow/widower	6(1.9)
**Education level**	No formal education	53(16.8)
	Primary level	169(53.7)
	Secondary level	62(19.7)
	College level	14(4.4)
	University/Tertiary level	17(5.4)
**Place of residence**	Rural area	175(55.6)
	Urban area	140(44.4)
**Employment status**	Government employee	26(8.3)
	Private sector employee	18(5.7)
	Self employed	188(59.7)
	Non-employed	83(26.3)
	Government insurance scheme eg NHIF	50(15.9)

### History of exposure to risk factors for head and neck cancer among respondents (*N* = 315)

Majority of the respondents, 295(93.7%) were non-smokers and the least number of study participants were former smokers, 15(4.7%). Regarding history of orogenital sexual practice, most respondents had no prior history of orogenital sexual practice 314(99.7%). Similarly, 245(77.8%) respondents had no history of alcohol consumption and all the respondents had no prior history of chewing/sniffing tobacco (Table 2).

**Table 2:** History of exposure to risk factors for head and neck cancers among respondents

**Variable**	**Characteristic**	**Frequency, N(%)**
**Tobacco use/smoking**	None smoker	295(93.7)
	Current smoker	5(1.6)
	Former smoker	15(4.7)
**History of orogenital sexual practice**	Yes	1(0.3)
	No	314(99.7)
**History of chewing/sniffing tobacco**	Yes	0(0)
	No	315(100.0)
**History of alcohol consumption**	Yes	70(22.2)
	No	245(77.8)

### Awareness on head and neck cancer among the study participants

- (i) Whether one considers himself/herself to be knowledgeable on head and neck cancer

In this study, 173(54.9%) respondents considered themselves to be somewhat knowledgeable on head and neck cancer while 9(2.9%) considered themselves to be extremely knowledgeable on head and neck cancer (Table 3).

- (ii) Anatomical sites of head and neck cancer identified by respondents

**Table 3:** Whether one considers himself/herself to be knowledgeable on head and neck cancers

	**Characteristic**	**Frequency, N(%)**
**Do you consider yourself to be knowledgeable/aware on Head and Neck Cancers?**	Not at all knowledgeable	97(30.8)
	Somewhat knowledgeable	173(54.9)
	Very knowledgeable	36(11.4)
	Extremely knowledgeable	9(2.9)
	Total	315(100.0)

**Fig. 1:**
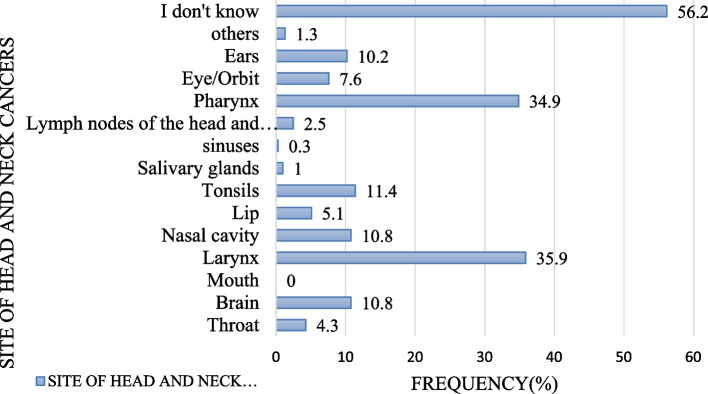
Anatomical sites of head and neck cancers identified by respondents

Majority of the respondents, 177(56.2%) didn't knew anatomical sites of head and neck cancer and 4(1.3%) respondents incorrectly identified others anatomical sites of HNCs (like lung, chest and heart). Similarly, 34(10.8%) respondents incorrectly identified brain as a subsite of HNCs, 113(35.9%) respondents correctly knew larynx as another subsite while sinuses (0.3%) were the least correctly known subsites of HNCs (Fig. 1).

- (iii) Signs and symptoms of head and neck cancer identified by respondents

**Fig. 2:**
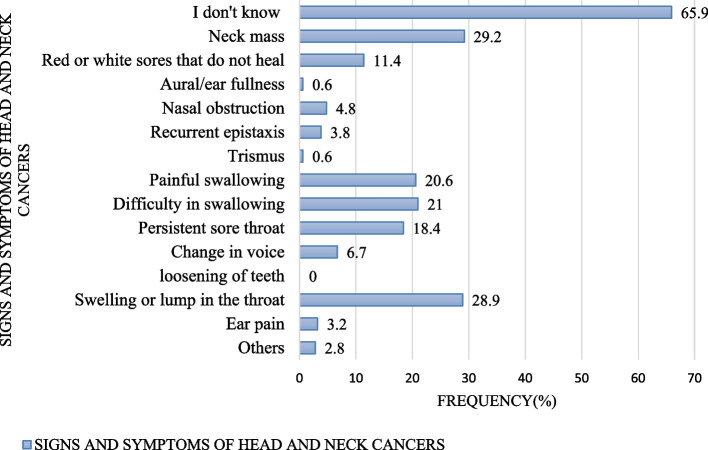
Signs and symptoms of head and neck cancers identified by respondents

In this study, 208(65.9%) respondents didn't know signs and symptoms of head and neck cancer and 9(2.8%) respondents incorrectly identified other nonspecific symptoms for HNCs (like chronic cough, blood in sputum, difficulty in breathing, loss of appetite, fever, persistent headache and poor vision). In the same study, neck mass was the most correctly identified symptom for HNCs, 92 (29.2%) while loosening of teeth was unknown to all respondents (Fig. 2).

- (iv) Risk factors for head and neck cancer identified by respondents

**Fig. 3:**
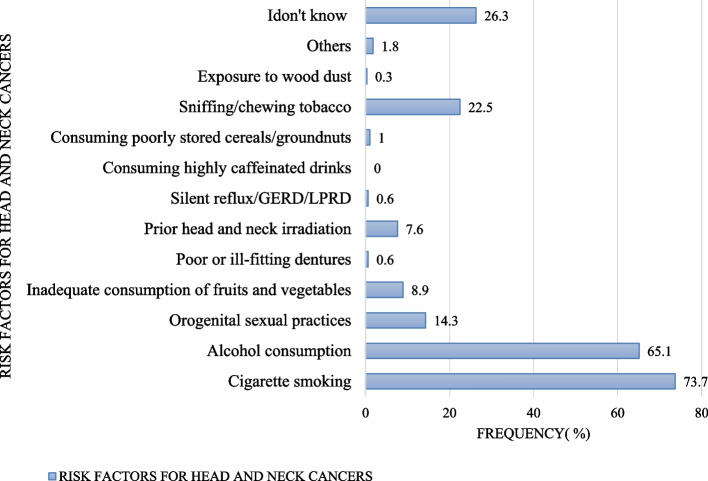
Risk factors for head and neck cancers identified by respondents

Majority of the respondents, 232(73.7%) knew cigarette smoking as a risk factor for head and neck cancer. Alcohol consumption was the second most commonly known risk factor, 205(65.1%). Similarly, 6(1.8%) respondents reported others risk factors for HNCs (like exposure to any chemical, drug abuse, multiple sexual partners and exposure to sugar containing foods) and 83(26.3%) respondents didn't knew the risk factors for HNCs (Fig. 3).

- (v) Awareness on curability and vaccines availability for prevention of head and neck cancer

The study has found that, 259(82.2%) respondents knew head and neck cancer to be potentially curable if diagnosed at early stages. Regarding vaccines, majority of the respondents were uncertain about its uses in preventing HNCs, 273(86.7%).

- (vi) Treatment options for head and neck cancer identified by respondents

Majority of the respondents, 289(91.7%) knew surgery as the treatment option for head and neck cancer and the least known treatment option was immunotherapy, 5(1.6%). In the same study, 20(6.3%) thought cancer to have no treatment.

- (vii) Preventive measures for head and neck cancer identified by respondents

**Fig. 4:**
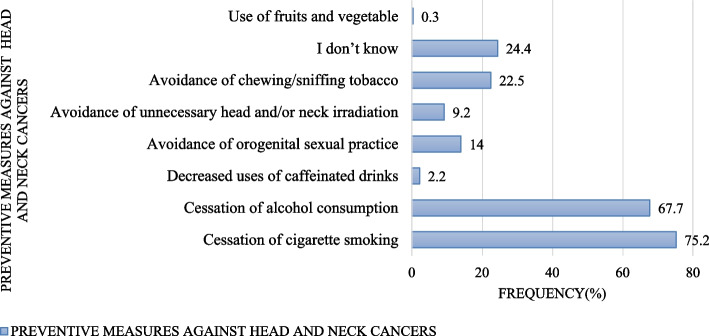
Preventive measures for head and neck cancers identified by respondents

Majority of the respondents, 237(75.2%) knew cessation of cigarette smoking to be a preventive measure for head and neck cancer. Cessation of alcohol consumption was the second most commonly known preventive measure for HNCs, 213(67.6%) while use of fruits and vegetable was the least known preventive measure, 1(0.3%). In the same study, 77(24.4%) respondents didn’t know preventive measures for HNCs (Fig. 4).

### Association between socio-demographic characteristics of the respondents and correctly identified alcohol consumption as a risk factor for head and neck cancer

This study has found a significant association between correctly identified alcohol consumption as a risk factor for head and neck cancer and some socio-demographic characteristics of the study participants like gender, level of education and marital status (their corresponding p-values being less than 0.05). In the same study, no association was found between correctly identified alcohol consumption as a risk factor for head and neck cancer and respondent’s age (*p* value = 0.826) (Table 4).

**Table 4:** Association between socio-demographic characteristics of study participants and correctly identified alcohol consumption, cigarette smoking, chewing/sniffing tobacco as risk factors for head and neck cancers

**Variable**	**Characteristics**	**Alcohol consumption N(%)**	**Cigarette smoking N(%)**	***Sniffing/chewing tobacco, N(%)***
**Sex**	Male	78(73.6)	88(83.0)	32(30.2)
	Female	127(60.8)	144(68.9)	39(18.7)
**Age**	18–33	110(66.3)	121(72.9)	46(27.7)
	34–49	62(62.6)	75(75.8)	17(17.2)
	50 and above	33(66.0)	36(72.0)	8(16.0)
**Marital status**	Single	30(83.3)	34(94.4)	14(38.9)
	Married	162(63.3)	182(71.1)	54(21.1)
	Divorced	11(64.7)	14(82.4)	3(17.6)
	Widow/widowed	2(33.3)	2((33.3)	0(0.0)
**Education level**	No formal education	29(54.7)	35(66%)	8(15.1)
	Primary level	104(61.5)	120(71.0)	24(14.2)
	Secondary level	44(71.0)	47(75.8)	19(30.6)
	College level	13(92.9)	14((100)	10(71.4)
	University level	15(88.2)	16(94.1)	10(58.8)

### Association between socio-demographic characteristics of respondents and correctly identified cigarette smoking as a risk factor for head and neck cancer

The study has found a statistically significant association between correctly identified cigarette smoking as a risk factor for head and neck cancer and some of the socio-demographic characteristics of the respondents (like gender, level of education and marital status) since all their corresponding p-values are less than 0.05 though no association was found between correctly identified cigarette smoking as a risk factor for head and neck cancer and respondent’s age (*p* value = 0.826) (Table 4).

### Association between socio-demographic characteristics of respondents and correctly identified chewing/sniffing tobacco as a risk factor for head and neck cancer

The study has found a statistically significant association between correctly identified chewing/sniffing tobacco as a risk factor for head and neck cancer and some of the socio-demographic characteristics of the respondents (like gender, level of education and marital status) since their corresponding p-values are less than 0.05 though no association was found between correctly identified chewing/sniffing tobacco as a risk factor for head and neck cancer and respondent’s age (*p* value = 0.826) (Table 4).

### Association between respondent’s risk factor status and correctly identified risk factor for head and neck cancer

The study has found no statistically significant association between respondent’s tobacco use/smoking status and correctly identified tobacco use/smoking as a risk factor for head and neck cancer (*p* value = 0.064). In the same study, a statistically significant association between respondent’s alcohol consumption status and correctly identified alcohol consumption as a risk factor for head and neck cancer was found (*p*-values = 0.07) (Table 5).

**Table 5:** Association between respondent’s risk factor status and correctly identified risk factor for head and neck cancers

	**Characteristics**	**Cigarette smoking, N (%)**	**Total N(%)**	***p*-value**
**History of tobacco use/smoking**	Non smoker	213(72.2)	295(100)	0.064
	Current smoker	5(100)	5(100)	
	Former smoker	14(93.3)	15(100)	
**History of alcohol consumption**		**Alcohol consumption, N(%)**		0.007
	Yes	36(51.4)	70(100)	
	No	169(69.0)	245(100)	

## Discussion

Most cases of head and neck cancer are diagnosed at later stages owing to various factors. A common reason for delayed diagnosis is that patients cannot identify the early signs and symptoms of HNCs. Therefore, improving awareness of the general population on HNCs will improve cancer prognosis since it enables early diagnosis to be made. To the best of our knowledge this is the first study in Tanzania to explore awareness of head and neck cancer.

In this study, 54.9% of the respondents considered themselves to be somewhat knowledgeable on HNCs. Such finding appear to be in line with what was found in the study that was conducted in Saudi Arabia where 49.3% of the study participants did not consider themselves to be knowledgeable on HNCs [13] but dissimilar to what was found in the United States of America where 66% of the study participants reported to be “not very” or “not at all” knowledgeable on HNCs [9].

Our study revealed that there was insufficient awareness on identification of the anatomical sites for HNCs**.** Over half of the respondents (56.2%) didn't know the anatomical sites of HNCs, 10.8% incorrectly identified the brain as a subsite HNCs and fewer respondents correctly identified some of the common subsites such as larynx (35.9%) and pharynx (34.9%). Such findings appear to be in line with what was found in the study from the United States where 21.0% of the respondents incorrectly identified the brain as a subsite of HNCs and only 2.0% of the respondents correctly identified the larynx as a subsite of HNCs [9].

Regarding signs and symptoms of HNCs, 65.9% of our study respondents didn't know signs and symptoms of HNCs and few respondents correctly identified some of the signs and symptoms of HNCs such as neck mass (29.2%), swelling or lump on the throat (28.9%), red or white sores that do not heal (11.4%) and persistent sore throat (18.4%). Such findings appear to be similar to what has been reported from the study that was conducted in the United States where only 15% of the study participants recognized “red or white sores that do not heal” and less than 5% recognized other important symptoms such as neck swelling or lumps in the throat as symptoms for HNCs [9].

The same finding appear to resemble those from Saudi Arabia where most participants identified lump in the neck (50%), red or white sores that do not heal (30%), swelling or lump in the throat (40%), sore throat (20%), ear pain (20%), change in voice (25%), loosening of teeth (85%) [13].

Pertaining awareness on risk factors for head and neck cancer, our study found cigarette smoking (73.7%) and alcohol consumption (65.1%) to be the most identified potential risk factors by our respondents while few were aware of other risk factors such as orogenital sexual practices, inadequate consumption of fruits and vegetables, prior head and neck irradiation and sniffing/chewing tobacco. Such findings correlate with those from Saudi Arabia where alcohol and tobacco use were similarly identified as potential risk factors for HNCs by most of the study participants [13].

Regarding the proportion of study participants who doesn’t know the potential risk factors for HNCs, our study found 26.3% of the respondents to be well informed on the potential risk factors for HNCs. This finding appear to be dissimilar to what was found in the study that was conducted in the United States of America where 45.5% of the respondents had inadequate awareness on such risk factors [9]. On the other hand, essentially 100% of the respondents were non-smokers, both current and former non-smoking groups and since this figure doesn't correspond to the prevalence of smoking among normal population of Geita region in Tanzania then this could be due to respondent’s reveal bias.

Although most of the respondents knew HNCs to be potentially curable if diagnosed at early stages, little is known about the treatment options for HNCs. Only surgery was the most known treatment option for HNCs (91.7%) with an alarming number of subjects who had little awareness on other treatment options such as radiotherapy, chemoradiation and immunotherapy. Perhaps surgery as the commonest known treatment modality for head and neck cancer by patients in our study may be a local myth that may have been prevailing within the studied population or may be a wrong option to the studied patients.

Most of the respondents in our study had good awareness on cessation of cigarette smoking and alcohol consumption as preventive measures for HNCs. Other preventive measures of HNCs were less known to most of the respondents.

Pertaining the association between socio-demographic characteristics of respondents and correctly identified risk factors for HNCs, our study has found a significant association between the overall awareness on correctly identified risk factors (like cigarette smoking, alcohol consumption and sniffing/chewing tobacco) and some socio-demographic characteristics of the respondents like gender, marital status and level of education (their corresponding p-values being less than 0.05) though no association was found between correctly identified risk factors and age (p value = 0.826). These results were consistent with those obtained from the United States of America [9] but dissimilar to those from Saudi Arabia where there was a strong association between awareness on some risk factors for HNCs and some socio-demographic characteristics such as tobacco and alcohol consumption [13].

Regarding association between respondent’s risk factor status and correctly identified risk factors for HNCs, this study has found no significant association between respondent’s cigarette smoking status and awareness on cigarette smoking as a risk factor for HNCs (*p* value = 0.064). This finding appear dissimilar to what was found in the United States [9]. Similarly**,** this study found a significant association between respondent’s alcohol consumption status and awareness on alcohol consumption as a risk factor for head and neck cancer (*p* value = 0.007) that is also dissimilar to what was found in the United States [9].

The study has a limitation of being from a single regional referral hospital in Tanzania and involving a smaller sample size and therefore the findings are not generalizable. Some information like percentage of patients from outpatients or inpatients or specified patients from certain specialty clinics wasn’t collected and therefore difficult for subsequent analysis to be done to ascertain which group is less aware of head and neck cancers than their counterparts.

## Conclusion and recommendations

Although majority of the respondents considered themselves to be somewhat knowledgeable on head and neck cancer on self-perceived basis, more than half of the respondents didn’t know the anatomical sites for head and neck cancer and similar estimate could not identify the symptoms for such cancer. About two-third knew cigarette smoking to be the commonest risk factor for head and neck cancer. More than two-third knew head and neck cancer to be potentially curable if diagnosed at early stages and also knew surgery to be the treatment of choice for such cancers. Moreover, community educational interventions should be implemented though health promotions and educational campaigns to increase awareness on head and neck cancer in this era of rampant mortality due to non-communicable diseases. Larger multicentric studies and also community-based studies are highly recommended so as to assess awareness of head and neck cancer among Tanzanian’s since it’s of paramount importance in this era of emerging head and neck cancers.

## Data Availability

The datasets generated during and/or analyzed during the current study are available from the corresponding author on reasonable request.

## Abbreviations

HNCs: Head and neck cancers
IRREC: Institutional Research Review Committee

## References

[1] Abraham ZS, Ntunaguzi D, Kahinga AA, Swai H, Mithe S, Massawe ER (2019). Clinico-pathological profile of paediatric head and neck cancers in Tanzania: findings from the country’s largest tertiary hospital. Int J Otorhinolaryngol Head Neck Surg.

[2] Abraham ZS, Kahinga AA, Swai H, Massawe ER (2018). Clinico-histocytopathological profile of paediatric head and neck malignant neoplasms: a mini-review. Med J Zambia.

[3] Dhull AK, Atri R, Dhankhar R, Chauhan AK, Kaushal V (2018). Major risk factors in head and neck cancer: a retrospective analysis of 12-year experiences. World journal of oncology.

[4] Mwansasu C, Liyombo E, Moshi N, Mpondo BC. Pattern of head and neck cancers among patients attending Muhimbili National Hospital Tanzania. Tanzania J Health Res. 2015;17(1):36–41.

[5] Gilyoma JM, Rambau PF, Masalu N, Kayange NM, Chalya PL (2015). Head and neck cancers: a clinico-pathological profile and management challenges in a resource-limited setting. BMC Res Notes.

[6] Abraham ZS, Massawe ER, Kahinga AA, Mapondella KB, Massawe WA, Swai H, Mithe S, Yahaya JJ, Ntunaguzi D (2020). Clinical profile of paediatric head and neck cancers at a tertiary hospital in Tanzania. Rwanda J Med Health Sci.

[7] DeSantis C, Naishadham D, Jemal A (2013). Cancer statistics for African Americans, 2013. CA Cancer J Clin.

[8] Siegel R, Naishadham D, Jemal A (2013). Cancer statistics, 2013. CA Cancer J Clin.

[9] Luryi AL, Yarbrough WG, Niccolai LM, Roser S, Reed SG, Nathan CA, Moore MG, Day T, Judson BL (2014). Public awareness of head and neck cancers: a cross-sectional survey. JAMA Otolaryngology-Head Neck Surg.

[10] Mehanna H, Paleri V, West CM, Nutting C (2010). Head and neck cancer—Part 1: Epidemiology, presentation, and prevention. BMJ.

[11] Leoncini E, Ricciardi W, Cadoni G, Arzani D, Petrelli L, Paludetti G, Brennan P, Luce D, Stucker I, Matsuo K, Talamini R (2014). Adult height and head and neck cancer: a pooled analysis within the INHANCE Consortium. Eur J Epidemiol.

[12] Dubai SA, Ganasegeran K, Alabsi AM, Alshagga MA, Ali RS (2012). Awareness and knowledge of oral cancer among university students in Malaysia. Asian Pac J Cancer Prev.

[13] Alqaryan S, Aldrees T, Almatrafi S, Alharbi A, Alhumaid H (2020). Awareness of head and neck cancers in Saudi Arabia: a questionnaire based study. Saudi Med J.

[14] Patton LL, Agans R, Elter JR, Southerland JH, Strauss RP, Kalsbeek WD (2004). Oral cancer knowledge and examination experiences among North Carolina adults. J Public Health Dent.

[15] Osazuwa-Peters N, Adjei Boakye E, Hussaini AS, Sujijantarat N, Ganesh RN, Snider M, Thompson D, Varvares MA (2017). Characteristics and predictors of oral cancer knowledge in a predominantly African American community. PLoS One.

[16] Tomar SL, Logan HL (2005). Florida adults' oral cancer knowledge and examination experiences. J Public Health Dent.

[17] Warnakulasuriya KA, Harris CK, Scarrott DM, Watt R, Gelbier S, Peters TJ, Johnson NW (1999). An alarming lack of public awareness towards oral cancer. Br Dent J.

